# Demographic patterns in quantitative sensory testing and clinical pain among former professional American-style football players

**DOI:** 10.1097/PR9.0000000000001428

**Published:** 2026-04-03

**Authors:** Samantha M. Meints, Robert R. Edwards, Can Ozan Tan, Inana Dairi, Alicia J. Whittington, Julius Dewayne Thomas, Heather DiGregorio, Cheyenne Brown, Claudia M. Campbell, Elizabeth Nolan, Edgar Ross, Herman A. Taylor, Adam S. Tenforde, Meagan M. Wasfy, Marc Weisskopf, Ross Zafonte, Aaron L. Baggish, Rachel Grashow

**Affiliations:** a Department of Anesthesiology, Perioperative and Pain Medicine, Harvard Medical School, Brigham and Women's Hospital, Boston, MA, USA; b Department of Electrical Engineering, Mathematics, and Computer Science, RAM Group, University of Twente, the Netherlands; c Football Players Health Study at Harvard University, Harvard Medical School, Boston, MA, USA; d Department of Clinical Psychology, Nova Southeastern University, Fort Lauderdale, FL, USA; e Department of Environmental Health, Harvard T.H. Chan School of Public Health, Boston, MA, USA; f Department of Psychiatry and Behavioral Sciences, Johns Hopkins University School of Medicine, Baltimore, MD, USA; g Department of Orthopedics and Spine, Atrius Healthcare, Burlington, MA, USA; h Cardiovascular Performance Program, Massachusetts General Hospital and Harvard Medical School, Boston, MA, USA; i Department of Physical Medicine and Rehabilitation, Spaulding Rehabilitation Hospital, Charlestown, MA, USA; j Department of Physical Medicine and Rehabilitation, University of Missouri, Columbia, MO, USA; k Department of Cardiology, Lausanne University Hospital (CHUV) and Institute for Sport Science, University of Lausanne (ISSUL), Lausanne, Switzerland

**Keywords:** Chronic pain, Race, Age, Professional Athletes, quantitative sensory testing

## Abstract

**Introduction:**

Chronic pain is highly prevalent yet heterogeneous among former American-style football (ASF) players. Pain sensitivity, which differs by age and race, contributes to individual differences in clinical pain in the general population but has not been examined among elite athletes.

**Objectives:**

The aim of the current cross-sectional study was to (1) assess pain sensitivity using quantitative sensory testing (QST); (2) examine the impact of age and race on pain sensitivity; and (3) identify the extent to which pain sensitivity is associated with clinical pain intensity and whether this varies across age and race among former ASF players.

**Methods:**

Former players (N = 110; N_Black_ = 59; N_White_ = 51; M_age_ = 49) completed questionnaires and underwent QST.

**Results:**

Results of Wilcoxon rank-sum and Fisher exact tests indicate age- and race-related differences in QST whereby older and Black players reported greater painful temporal summation aftersensations (M_older_ = 2.89; SD_older_ = 6.89; M_Black_ = 18.03; SD_Black_ = 20.85) than younger and White players (M_younger_ = 0.32; SD_younger_ = 1.16; *P* < 0.01; M_White_ = 9.19; SD_White_ = 12.70; *P* = 0.03), and Black players demonstrated a lower tolerance for cold pain (M = 105.79; SD = 68.95) than White players (M = 144.66; SD = 57.15; *P* < 0.01). Results of multivariate linear and logistic regression models indicate the relationship between QST and clinical pain intensity differed by age and race. Greater cold pain tolerance was associated with less clinical pain but only for older players. Race differences in the relationship between QST and clinical pain varied across pain modalities.

**Conclusion:**

Results demonstrate that age- and race-related pain disparities persist among former ASF players. Moreover, age and race differences in pain sensitivity may be contributing to these disparities.

## Abstract 2

Supplemental Digital Content is Available in the Text.

Despite overall hyposensitivity, there are race- and age-related differences in the relationships between clinical and laboratory-based pain among former professional American-style football players.

## 1. Introduction

Chronic pain is highly prevalent and costly.^4,9,13,36,57^ Sports injuries, common among elite athletes including American-style football (ASF) players, result in acute and chronic pain.^25,41,55^ Although chronic pain among former ASF players is common, there are individual differences in the pain experience.

The biopsychosocial model posits that the development and maintenance of chronic pain is determined by complex interactions between biological processes, psychosocial factors, and social determinants of health.^40^ Pain sensitivity, defined as individual differences in pain perception (ie, nociception), is a biological mechanism that contributes to the pain experience. Quantitative sensory testing (QST) quantifies a person's somatosensory function, including peripheral and central pain sensitization, using calibrated, graded innocuous noxious stimuli (typically thermal and mechanical).^42^ Research has demonstrated that greater pain sensitivity is associated with greater pain severity and disability among people with chronic pain.^46^ However, it is unclear the extent to which differences in pain sensitivity contribute to pain outcomes among former ASF players, a population selected for their tolerance for pain and whose exposure to injury, psychosocial experiences, and socioeconomic status during their time of play may be very different than others in the general population.^60^

There are also demographic factors, including age and race, that influence the experience of pain. The burden of pain disproportionately affects members of racially minoritized groups, especially Black individuals,^30,47^ as well as older adults.^23,37,61^ Black individuals are more likely to experience pain and have more intense and disabling pain.^18^ The impact of age on pain is more nuanced such that older adults are more likely to experience pain and report greater pain–related dysfunction despite a lack of age-related differences in pain intensity.^53,56^

In addition to age- and race-related differences in clinical pain, there are also age and race differences in pain sensitivity. Although the results vary based on the body location, pain stimulus, and type of assessment (eg, threshold, tolerance, etc.), studies have shown age-related differences in pain sensitivity. For example, older adults are less sensitive to warmth and heat pain,^54^ have a decline in pain inhibition (ie, conditioned pain modulation),^11,52^ and show a general increase in pain thresholds.^28^ The results are more consistent for race differences in experimental pain where Black individuals demonstrate greater sensitivity to pain across body location, stimulus, and type of assessment.^30,32,34,35^ Similar to what has been found in the general population, it is possible that the age and race differences we see in pain sensitivity contribute to differences in clinical pain. However, this has yet to be examined among elite athletes such as former ASF players.

The aim of the current study was to (1) assess pain sensitivity among former ASF players using QST; (2) examine the impact of age and race on pain sensitivity; and (3) identify the extent to which pain sensitivity is associated with clinical pain intensity and whether this varies across age and race.

## 2. Methods

### 2.1. Design

The Football Players Health Study (FPHS) at Harvard University is a transdisciplinary, strategic initiative addressing health challenges of former ASF players.^60^ All aspects of this study were conducted at Harvard Medical School affiliated hospitals and were approved by the Mass General Brigham Human Subjects Research Committee (Protocol #2018P001929; NCT03866564).

### 2.2. Participants

From the FPHS parent study (a large-scale longitudinal cohort study of N = 4,189 former players; see Zafonte et al. 2019 for a detailed description of the FPHS parent study protocol), a subset of former players was invited to Boston to undergo an in-person, 3-day, comprehensive health assessment protocol designed to characterize and quantify various pathology.^8,60^ This cohort included players who identified either a single health condition or multiple health conditions across key domains of cardiometabolic disease, disordered sleep (sleep apnea), chronic pain (defined as self-reported use of medication prescribed by a health care provider for pain), and cognitive impairment (defined by having a dementia diagnosis or using medication for memory loss) along with players without reported health conditions who were matched for age and race. Players ages 29 to 55 who self-identified their race as either Black or White were enrolled. The present study includes only those participants (n = 110/111) who completed the QST protocol during the in-person assessment.

### 2.3. Measures

For the larger FPHS parent study, participants completed questionnaires that asked about age and self-reported race and ethnicity, and football variables including position played and number of years played. Physical activity, body mass index (BMI), and use of pain medications were also assessed at the time of the in-person study. Participants rated their current pain intensity at the time of the in-person assessment using a 0 to 10 numeric rating scale (NRS). The NRS is a valid and reliable assessment of pain intensity.^59^

The *International Physical Activity Questionnaire* was used to assess physical activity across the past week including job-related, transportation, housework, and recreational physical activity as well as time spent sitting.^3^ We calculated participants' moderate-to-vigorous physical activity using published standards^6^ and categorized them into meeting or not meeting US Health and Human Services recommendations (ie, ≥75 min/wk vigorous or ≥150 min/wk moderate or ≥120 min/wk moderate + vigorous activity).^48^

### 2.4. Quantitative sensory testing

#### 2.4.1. Cold pressor task

Cold pain was assessed using a cold pressor task (CPT). During this task, participants were asked to submerge their nondominant hand up to their wrist in a circulating bath of 4°C water (Thermo Scientific Arctic Series Refrigerated Bath Circulator, Thermo Scientific, Waltham, MA). They were instructed to leave their hand in the water until they could no longer tolerate the sensation. *Cold pain tolerance*, a measure of cold hyperalgesia, was recorded as the time at which participants withdrew their hand. There was a 3-minute limit on this task after which participants who had yet to remove their hand were asked to do so. Participants provided a pain rating (0–100 NRS) while their hand was in the water (20 seconds after placing their hand in the water; *cold pain rating (20 s), a measure of cold hyperalgesia*) and 30 seconds after removal of their hand from the water (*cold pain aftersensations, a measure of endogenous pain facilitation*). Because more than half of our sample reached the maximum cold pain tolerance time (3 minutes), we also created a dichotomous outcome variable for reaching the maximum tolerance time (max CPT reached).

#### 2.4.2. Temporal summation of mechanical punctate pain

*Temporal Summation* of Mechanical Punctate Pain (TSP), a dynamic test reflecting endogenous pain facilitation,^5^ was assessed using weighted pinprick stimulators.^33^ A 512-mN probe was applied to the skin on the dorsum of the right middle finger (middle phalanx) 10 times at a rate of 1 stimulus per second. Participants provided pain ratings using a 0 (no pain) to 100 (worst pain imaginable) NRS after the first and 10th stimulus application. To calculate TSP, the NRS rating after the first stimulus was subtracted from the NRS rating after the 10th stimulus. *Temporal summation aftersensations* were assessed 15 seconds after the 10th application using the 0 to 100 NRS.

#### 2.4.3. Pressure pain thresholds

*Pressure Pain Thresholds* (*PPTh;* an assessment of mechanical hyperalgesia) were assessed at the metacarpophalangeal joint of the thumb, the trapezius muscle, and the patella. Using a digital pressure algometer with a 0.785-cm^2^ probe (Wagner FDX, Greenwich, CT), pressure was applied and increased at a steady rate of 1Lb/second until the participant indicated the pressure was first perceived as painful.^31^ This was repeated 4 times at each site and averaged across the 4 trials.

### 2.5. Statistical analysis

We first explored descriptive statistics (means and standard deviations) for demographic, football, health, and pain variables for the total selected sample as well as for participants stratified by pain intensity ratings, age, and race. Pain ratings used for this stratification were provided before QST procedures during the in-person assessment and based on prior literature classifying numeric rating scale ratings 0 to 4 as mild-to-moderate and 5 to 10 as severe.^15^ Because chronic health conditions including painful osteoarthritis become more prevalent after age 45,^14^ we stratified our sample by age (those under 45 and those age 45 and older). Player race was self-identified as either Black or White. We then used nonparametric weighted Fisher exact test (categorial variables) and weighted Wilcoxon rank-sum tests (continuous variables) to examine univariate differences by pain strata, age, and race. *P*-values were determined based on unweighted data.

Because several QST variables were not normally distributed, we dichotomized the variables for use in multivariate models. Specifically, there were floor effects for temporal summation and temporal summation after sensations with 59 and 82 participants providing an NRS rating of “0,” respectively. There were ceiling effects observed for the pressure pain thresholds at each anatomical site with 65, 72, and 90 participants reaching the maximum pressure of 20 pounds of force at the thumb, trapezius, and knee, respectively. As a result, we dichotomized these variables as experiencing temporal summation (TS > 0) or not; experiencing temporal summation after sensations (NRS > 0) or not (NRS = 0); and reaching maximum PPTh (PPTh > 20 pounds of force) or not (PPTh < 20). In addition, cold pain aftersensations were dichotomized using a median split (Mdn = 7.5). We used backwards selection in weighted multivariate linear and logistic regression models to separately examine the influence of age and race on pain intensity and QST variables (ie, cold pain tolerance time [CPTo], cold pain rating [20 seconds], cold pain aftersensations, maximum CPT reached, temporal summation [TS], temporal summation aftersensations, and pressure pain threshold [PPTh] at the thumb, trapezius, and knee) that were additionally adjusted for BMI,^43^ linemen status (linemen are at greater risk for pain comorbidities),^10,16^ number of surgeries (a proxy for number of serious injuries which are associated with pain^21,24^), concussion symptom history,^38^ use of pain medication,^62^ and physical activity (associated with QST and clinical pain^2,44^), all of which are shown to be associated with pain processing including QST and/or clinical pain ratings. It is important to note that although these covariates were initially controlled for in the models, only variables that were significant predictors were kept in the final models. To address potential bias from selection from the parent into the in-person study, we calculated stabilized inverse probability weights for inclusion and used these weights in all multivariable models (see Supplemental Methods, supplemental digital content, http://links.lww.com/PR9/A395).^7,19,20^

In a second series of multivariate linear regression models, we investigated the relationship between clinical pain intensity (dependent variable) and QST-based pain sensitivity variables (as the independent variable in separate models). These weighted multivariable models adjusted for the health and football variables described above and additionally included interaction terms of pain sensitivity variables with age and race. R Language for Statistical Computing (Version 4.2^50^) was used to conduct all statistical analyses. Effects were considered significant at the *P* < 0.05 level.

## 3. Results

Of the 110 former ASF players who participated in the in-person health assessment with lab and subjective pain data and who were recruited based on health status, 51 (46%) self-identified as White and 59 (54%) self-identified as Black (Table 1). Participants were middle-aged (49 [SD = 8] years old) and played ASF for an average of 6 (SD = 3) years. In unweighted bivariable analyses, there were significant differences between those reporting none-to-mild pain (n = 90; 82%) compared to those with moderate-to-severe pain (n = 20; 18%) such that those with moderate-to-severe pain reported greater pain intensity during the CPT (*P* = 0.03; d = 0.74) and demonstrated lower PPTh at the knee (*P* = 0.01; d = 0.78). There were also trending marginal differences such that those with moderate-to-severe pain played for more years (*P* = 0.06; d = 0.40), were less likely to achieve physical activity standards (*P* = 0.06; d = 0.18), and demonstrated a lower CPTo (*P* = 0.06; d = 0.53) (Table 1).

**Table 1:** Cohort characteristics stratified by pain intensity.

Characteristic	Overall	Mild clinical pain	Moderate to severe clinical pain	*P* *	Cohen d (95% CI)
	N = 110†	N = 90†	N = 20†		
Age	48.94 (7.87)	49.06 (8.10)	48.40 (6.86)	0.80	0.08 (−0.40, 0.57)
Age range				0.60	
<45	38 (35%)	32 (36%)	6 (30%)		
45+	72 (65%)	58 (64%)	14 (70%)		
Race				0.10	
White	51 (46%)	45 (50%)	6 (30%)		
Black	59 (54%)	45 (50%)	14 (70%)		
BMI	32.97 (5.79)	32.65 (5.56)	34.41 (6.68)	0.30	0.30 (−0.79, 0.18)
Years of professional play	5.58 (3.42)	5.33 (3.43)	6.70 (3.21)	0.06	0.40 (−0.89, 0.09)
Active and postcareer surgeries				0.40	0.19 (−0.67, 0.30)
0	17 (15%)	15 (17%)	2 (10%)		
1	35 (32%)	29 (32%)	6 (30%)		
2	26 (24%)	21 (23%)	5 (25%)		
3	20 (18%)	17 (19%)	3 (15%)		
4	8 (7.30%)	4 (4.40%)	4 (20%)		
5	3 (2.70%)	3 (3.30%)	0 (0%)		
8	1 (0.90%)	1 (1.10%)	0 (0%)		
Lineman	46 (42%)	39 (43%)	7 (35%)	0.50	
PA by USHHS criteria				0.06	
<USHHS guidelines	62 (56%)	47 (52%)	15 (75%)		
>USHHS guidelines	48 (44%)	43 (48%)	5 (25%)		
Pain medications	26 (24%)	16 (18%)	10 (50%)	0.01	
Pain interference (t-score)	53.81 (9.25)	51.63 (7.98)	64.96 (7.08)	<0.01	1.70 (−2.26, −1.12)
Missing	6	3	3		
Clinical pain intensity	1.88 (2.20)	1.00 (1.08)	5.85 (1.46)	<0.01	4.19 (−4.93, −3.45)
Cold pain tolerance (CPTo)	124.82 (66.07)	131.23 (63.39)	97.07 (72.01)	0.06	0.53 (0.01, 1.04)
Missing	14	12	2		
Maximum CPTo reached				0.12	
Sub-max	43 (45%)	32 (41%)	11 (61%)		
Max	53 (55%)	46 (59%)	7 (39%)		
Missing	14	12	2		
Cold pain rating	36.68 (28.12)	33.23 (25.64)	53.25 (34.15)	0.03	0.74 (−1.28, −0.18)
Missing	17	13	4		
Cold pain aftersensations	13.70 (17.82)	12.57 (17.33)	18.86 (19.58)	0.20	0.35 (−0.87, 0.16)
Missing	10	8	2		
Temporal summation	5.45 (12.10)	6.09 (13.00)	2.65 (6.29)	0.20	0.28 (−0.20, 0.77)
Missing	2	2	0		
Temporal summation aftersensations	1.99 (5.72)	1.99 (5.48)	2.00 (6.93)	0.40	0.00 (−0.50, 0.49)
Missing	1	0	1		
PPTh at thumb	18.04 (3.56)	18.35 (3.16)	16.68 (4.81)	0.05	0.48 (−0.01, 0.96)
Missing	2	2	0		
PPTh at trapezius	18.47 (3.06)	18.81 (2.55)	16.98 (4.47)	0.20	0.61 (0.12, 1.11)
Missing	3	3	0		
PPTh at knee	19.44 (1.83)	19.70 (1.32)	18.33 (3.02)	0.01	0.78 (0.28, 1.27)
Missing	3	3	0		

* Wilcoxon rank-sum test; Pearson χ^2^ test; Fisher exact test.

† Mean (SD); n (%).

PA, physical activity; USHHS, United States Department of Health and Human Services; CPTo, cold pain tolerance in second; PPTh, pressure pain threshold.

In unweighted analyses exploring age differences in clinical pain intensity and pain sensitivity, we found that older former players experienced greater aftersensations after the TS task (*P* < 0.01; d = 0.46; Table 2). However, there were no other age-related differences in this sample. When participants were stratified by race in bivariable unweighted analyses, we identified significant differences between Black and White former players for CPTo (*P* < 0.01; d = 0.61), reaching the maximum CPTo time (*P* = 0.01), cold pain ratings (*P* < 0.01; d = 0.61), and cold pain aftersensations (*P* = 0.03; d = 0.51) along with marginal differences in PPTh at the thumb (*P* = 0.06; d = 0.31) such that Black players demonstrated greater sensitivity than White players (Table 3). Black players left their hands in the water for an average of 106 seconds (SD = 69) including 43% who reached the maximum tolerance time of 3 minutes. They rated their pain as 45/100 (SD = 30) during the task and 18/100 (SD = 21) 30 seconds after removing their hand. White players left their hands in the water for an average of 145 seconds (SD = 57) including 68% who reached the maximum tolerance time. They rated their pain as 29/100 (SD = 24) during the task and 9/100 (SD = 13) after removing their hands. Black players reached PPTh at the thumb at 17.5 (SD = 4.1) pounds of force, whereas White players reached PPTh at 18.6 (SD = 2.8) pounds of force.

**Table 2:** Unweighted descriptive statistics for pain outcomes stratified by age.

Characteristic	Overall	<45	45+	*P* *	Cohen d (95% CI)
	N = 110†	N = 38†	N = 72†		
Pain interference (t-score)	53.81 (9.25)	53.69 (9.86)	53.87 (8.98)	0.80	0.02 (−0.42, 0.38)
Missing	6	2	4		
Clinical pain intensity	1.88 (2.20)	1.66 (2.11)	2.00 (2.26)	0.40	0.15 (−0.55, 0.24)
Clinical pain (categorical)				0.60	
None-to-mild clinical pain	90 (82%)	32 (84%)	58 (81%)		
Moderate-to-severe clinical pain	20 (18%)	6 (16%)	14 (19%)		
Cold pain tolerance (CPTo)	124.82 (66.07)	128.28 (67.14)	123.09 (65.99)	>0.90	0.08 (−0.35, 0.50)
Missing	14	6	8		
Maximum CPTo reached				0.90	
Max	43 (45%)	14 (44%)	29 (45%)		
Sub-max	53 (55%)	18 (56%)	35 (55%)		
Missing	14	6	8		
Cold pain rating	36.68 (28.12)	36.27 (29.12)	36.90 (27.81)	0.70	0.02 (−0.45, 0.40)
Missing	17	5	12		
Cold pain aftersensations	13.70 (17.82)	12.16 (14.47)	14.49 (19.38)	>0.90	0.13 (−0.54, 0.28)
Missing	10	4	6		
Temporal summation	5.45 (12.10)	6.76 (13.78)	4.77 (11.17)	0.60	0.16 (−0.23, 0.56)
Missing	2	1	1		
Temporal summation aftersensations	1.99 (5.72)	0.32 (1.16)	2.89 (6.89)	0.003	0.46 (−0.86, −0.06)
Missing	1	0	1		
PPTh at thumb	18.04 (3.56)	18.51 (3.37)	17.80 (3.65)	0.14	0.20 (−0.20, 0.60)
Missing	2	1	1		
PPTh at trapezius	18.47 (3.06)	18.94 (2.41)	18.23 (3.33)	0.30	0.23 (−0.17, 0.63)
Missing	3	2	1		
PPTh at knee	19.44 (1.83)	19.50 (1.84)	19.41 (1.84)	0.40	0.05 (−0.35, 0.45)
Missing	3	2	1		

* Unweighted Wilcoxon rank-sum test; unweighted Fisher exact test.

† Mean (SD); n (%).

CPTo, cold pain tolerance in second; PPTh, pressure pain threshold.

**Table 3:** Unweighted descriptive statistics for pain outcomes stratified by race.

Characteristic	Overall	White	Black	*P* *	Cohen d (95% CI)
	N = 110†	N = 51†	N = 59†		
Pain interference (t-score)	53.81 (9.25)	54.81 (7.62)	52.92 (10.48)	0.20	0.20 (−0.18, 0.59)
Missing	6	2	4		
Clinical pain intensity	1.88 (2.20)	1.84 (1.82)	1.92 (2.51)	0.30	−0.03 (−0.41, 0.34)
Clinical pain (categorical)				0.10	
None-to-mild clinical pain	90 (82%)	45 (88%)	45 (76%)		
Moderate-to-severe clinical pain	20 (18%)	6 (12%)	14 (24%)		
Cold pain tolerance (CPTo)	124.82 (66.07)	144.66 (57.15)	105.79 (68.95)	<0.01	0.61 (0.20, 1.02)
Missing	14	4	10		
Maximum CPTo reached				0.01	
Sub-max	43 (45%)	15 (32%)	28 (57%)		
Max	53 (55%)	32 (68%)	21 (43%)		
Missing	14	4	10		
Cold pain rating	36.68 (28.12)	28.73 (23.81)	45.16 (30.09)	0.01	0.61 (−1.02, −0.19)
Missing	17	3	14		
Cold pain aftersensations	13.70 (17.82)	9.19 (12.70)	18.03 (20.85)	0.03	0.51 (−0.91, −0.11)
Missing	10	2	8		
Temporal summation	5.45 (12.10)	3.82 (8.93)	6.91 (14.28)	0.20	0.26 (−0.64, 0.12)
Missing	2	0	2		
Temporal summation aftersensations	1.99 (5.72)	2.35 (6.37)	1.67 (5.12)	0.60	0.12 (−0.26, 0.49)
Missing	1	0	1		
PPTh at thumb	18.04 (3.56)	18.62 (2.76)	17.52 (4.10)	0.06	0.31 (−0.07, 0.69)
Missing	2	0	2		
PPTh at trapezius	18.47 (3.06)	19.05 (2.27)	17.94 (3.57)	0.20	0.37 (−0.02, 0.75)
Missing	3	0	3		
PPTh at knee	19.44 (1.83)	19.74 (0.96)	19.17 (2.33)	0.50	0.31 (−0.07, 0.69)
Missing	3	0	3		

* Unweighted Wilcoxon rank-sum test; unweighted Fisher exact test.

† Mean (SD); n (%).

CPTo, cold pain tolerance in second; PPTh, pressure pain threshold.

Results of multivariate models examining the effects of age and race on clinical pain and QST indicate that age was significantly associated with the TS aftersensations and PPTh at the thumb such that older players were more likely to report painful TS aftersensations (OR = 0.21, *P* < 0.05) and more likely to have PPTh at the thumb less than 20 pounds of force (OR = 0.18, *P* < 0.01; Table 4). In these models, self-identifying as Black was associated with greater clinical pain intensity (β = 1.2; *P* < 0.01), lower CPTo (β = −70, *P* < 0.01), less likelihood of reaching the maximum CPTo time (OR = 0.14, *P* < 0.01), greater cold pain ratings (β = 10, *P* = 0.08), and less likelihood of reaching a PPTh at the thumb less of 20 pounds of force (OR = 0.17, *P* < 0.01; Table 4).

**Table 4:** Weighed estimated coefficients for the race and age variables in models predicting the dependent variable listed in column 1.

	Age term estimate 45+ Est. (95% C.I.), *P*	Race term estimate Black Est. (95% C.I.), *P*
Clinical pain intensity		β = 1.2 (0.37 to 2), <0.01
CPTo time	β = −21 (−47 to 5), 0.11	β = −70 (−95 to −45), <0.01
Max CPTo reached		OR = 0.14 (0.04 to 0.39), <0.01
Cold pain rating		β = 10 (−1.3 to 22), 0.08
Cold pain aftersensations		
Temporal summation		
Temporal summation aftersensations	OR = 0.21 (0.04 to 0.72) 0.02	
PPTh—thumb	OR = 0.18 (0.05 to 0.53), <0.01	OR = 0.17 (0.05 to 0.51), <0.01
PPTh—trapezius		OR = 0.42 (0.15 to 1.15), 0.10
PPTh—knee		

Odds ratios (OR) are shown for pain categorical outcomes; β are shown for continuous pain outcomes. All models were adjusted for age, race, BMI, lineman status, number of surgeries, CSS burden, pain medications, and physical activity whenever applicable. Age under 45, White race, non-lineman status, not meeting physical activity standards, and no pain medication reporting served as reference.

CPTo, cold pain tolerance in seconds; PPTh, pressure pain threshold.

**Figure 1.**
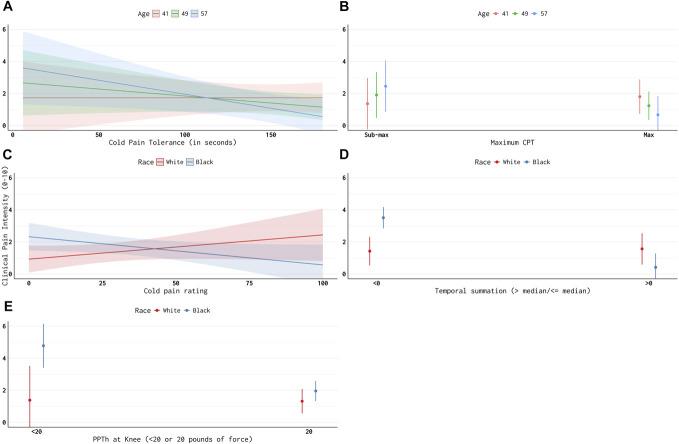
Relationships between reported clinical pain intensity and quantitative sensory testing, accounting for age and race.

Finally, we investigated the relationship between clinical pain and QST pain measures and the extent to which these relationships differed by age and race. We found that CPTo (β = 0.046, *P* = 0.06) and reaching maximum CPTo time (β = 6.3, *P* = 0.05) were marginally associated with lower clinical pain intensity (Table 5). Relationships between clinical pain and CPTo time and likelihood of reaching maximum CPTo time differed significantly by age such that greater CPTo and reaching maximum CPTo time were associated with less clinical pain particularly among older players (Table 5, Figs. 1A, B). The relationship between clinical pain and cold pain ratings, temporal summation of pain, and PPth at the knee differed significantly by race (Table 5). Greater cold pain ratings were associated with greater clinical pain among White players, whereas greater cold pain ratings were associated with less clinical pain among Black players (Fig. 1C). Experiencing temporal summation of pain was associated with less clinical pain among Black players, but there was no relationship between temporal summation and clinical pain among White players (Fig. 1D). Having a PPTh at the knee less than 20 pounds of force was associated with greater clinical pain but only for Black players (Fig. 1E).

**Table 5:** Estimated coefficients and variation explained in the weighted linear relation between subjective (reported clinical pain intensity) and objective measures of pain, accounting for age and race.

Pain outcome	Clinical pain intensity term β (95% C.I.), *P*	Age × clinical pain outcome β (95% C.I.), *P*	Race × clinical pain outcome β (95% C.I.), *P*
Cold pain tolerance	**0.046 (0.00–0.09), 0.058**	**<0.01 (0.00–0.00), 0.02**	−0.01 (−0.03–0.01), 0.22
Maximum CPTo reached	**6.3** (−**0.09 to 13.00), 0.05**	**−0.14 (**−**0.27 to** −**0.02), 0.03**	−0.77 (−2.90–1.40), 0.47
Cold pain rating	−0.02 (−0.10–0.07), 0.66	<0.01 (0.00–0.00), 0.39	**−0.03 (**−**0.06 to 0.00), 0.02**
Cold pain aftersensations	−3.10 (−9.80 to 3.60), 0.36	0.08 (−0.05 to 0.21), 0.24	−1.20 (−3.20 to 0.77), 0.23
Temporal summation	−1.60 (−6.80 to 3.50), 0.53	0.04 (−0.07 to 0.14), 0.48	**−3.20 (**−**5.00 to** −**1.50), <0.01**
Temporal summation aftersensation	0.84 (−7.30 to 9.00), 0.84	−0.03 (−0.18 to 0.13), 0.73	2.00 (−0.28 to 4.30), 0.08
PPTh at thumb	−0.06 (−6.60 to 6.50), 0.99	−0.02 (−0.14 to 0.11), 0.77	−1.40 (−3.30 to 0.58), 0.17
PPTh at trapezius	−1.40 (−7.80 to 5.00), 0.67	0.01 (−0.11 to 0.14), 0.86	−0.92 (−2.90 to1.00), 0.35
PPTh at knee	7.40 (−3.80 to 19.00), 0.19	−0.16 (−0.36 to 0.05), 0.14	**−2.80 (**−**5.70 to 0.20), 0.07**

Bolded items indicate statistically significant or terending results at *p* < 0.10.

CPTo, Cold pain tolerance; PPTh, pressure pain threshold.

## 4. Discussion

The current study assessed pain sensitivity and clinical pain in former professional ASF players. Former ASF players demonstrate an overall hyposensitivity to pain across QST. For example, these players were incredibly tolerant of cold pain. Not only did more than half of players reach the maximum cold pressor tolerance time, but on average, their tolerance time was twice that of a sample of healthy, pain-free individuals who demonstrated an average cold pain tolerance of 67 seconds.^32^ Former players also demonstrated a low level of temporal summation of mechanical pain. Although players averaged 5 points of temporal summation, a previous study including both healthy, pain-free individuals and people with chronic low back pain indicated an average of 15 points of temporal summation using the same experimental paradigm.^33^ It is unclear whether their time as ASF players resulted in hyposensitivity to pain or whether an underlying trait of hyposensitivity to pain resulted in better football performance and thus likelihood of playing professional football. This hypothesis is consistent with data demonstrating that competitive athletes are less pain sensitive than normally active individuals.^58^ Such hyposensitivity (ie, hypoalgesia) is noteworthy and should be acknowledged when treating current and former ASF players as it can result in greater risk for injury. Pain serves as a critical warning signal that alerts an individual to potential or actual damage to the body and protects the body from further harm to promote healing. When this warning system is diminished, as in the case of hypoalgesia, individuals may not respond as quickly or appropriately to injurious situations leading to increased severity of injuries.^49^

Yet despite this seemingly exceptional response to pain, age and racialized disparities in the experience of pain seen in general populations persist among former ASF players. In the current study, compared to younger players (<45 years old), older players were more sensitive to QST tasks as demonstrated by being more likely to experience painful aftersensations after the TS task and to have a PPTh at their thumb of less than 20 pounds of force. The finding that endogenous pain facilitation, assessed with temporal summation, is enhanced among older adults in the general population^39^ was supported in our study of former players. On the other hand, our older players were less likely to reach a maximum PPTh of 20 pounds of force, which is inconsistent with prior work in the general population.^28^ It is possible that our age-related results differ from those in previous studies due to differences in study methodology. For example, it is common to assess PPTh at various body sites. In fact, in this study, we only found age-related differences in PPTh at the thumb but not trapezius or knee. It is possible that this was due to many of our participants reaching the ceiling of 20 pounds of force for the other more robust body sites. Together, this highlights the nuances in understanding pain perception and the need for consistent methodology across QST studies. In addition, there may be sample-related differences that account for different patterns of PPTh. Although the players in the current study were relatively young (our cut-off for older age was 45 years), this population experiences a premature burden of chronic disease and reduced healthspan.^17^ It remains unclear how this premature aging affects pain processing.

In the current study, Black players, compared to White players, reported a greater clinical pain intensity and were more sensitive to most QST assessments as evidenced by having a lower CPTo time and being less likely to reach the maximum CPTo time, and being less likely to reach a PPTh at the thumb of 20 pounds of force. These results are consistent with literature, which demonstrates robust racialized differences in cold pain and pressure pain sensitivity as assessed by QST across a wide range of individuals including those who are pain-free as well as those with chronic pain conditions.^26,27,32,35,51^ Likewise, these results are consistent with the race differences we observed in pain intensity among a larger sample of ASF players.^12^

Because race and age differences in pain are well documented^18,30,45^ and such documentation does not move the field toward ameliorating pain disparities,^22^ we aimed to better understand a potential mechanism of this disparity in this unique population. We examined the relationship between pain sensitivity and clinical pain as a potential mechanism for explaining identified age and race disparities in the pain experience among former ASF players. We found that less pain sensitivity (as measured by CPTo time and likelihood of reaching the max CPTo time) was associated with less clinical pain but only among older former players. Race-related findings were more variable. Although greater cold pain ratings during the CPT were associated with greater clinical pain intensity, as expected, this was only true for White players. Among Black players, greater cold pain ratings were associated with less clinical pain. Similarly, greater pain facilitation as assessed with by temporal summation of pain was associated with less clinical pain among Black players although there was no relationship among White players. On the other hand, having greater PPTh (ie, less sensitivity to mechanical pain) at the knee was associated with less clinical pain but only among Black players. Together, this suggests that there may be stronger relationships between pain sensitivity and clinical pain among Black but not White former players although a nuanced understanding is necessary as results vary based on assessment modality.

Several limitations should be considered when interpreting these findings. First, participants in the current study were recruited from the larger FPHS cohort and sampled based on race and health status.^8^ The current study included only a small subset (N = 110/3,995) of the total FPHS cohort. It is possible that the decision to participate, especially in the in-person assessment, was related to a participant's health and occupational flexibility in ways that were not captured by the selection weights, thus limiting the generalizability of these findings to those not represented in the in-person sample. Further, the study was not designed to evaluate the complex mechanisms that underlie pain disparities. Notably, race is inextricably tied to experiences with stress, structural and medical racism, and other biopsychosocial pressures, socioeconomic status, and other social determinants of health.^29^ However, we did not have information about players' current or past socioeconomic status. We were also limited in our analyses due to players' overall pain hyposensitivity and the resulting floor and ceiling effects of the data. It is possible that an alternative testing battery is necessary to better understand nociceptive processes among this population. Finally, we not only report significant findings (*P* < 0.05) but also trends nearing significance (*P* < 0.10). Further, consistent with recommendations, we did not apply corrections for multiple comparisons given that this was a secondary analysis and not the primary aim of the current study.^1^ Although this may inflate the likelihood of type I error, not reaching a significance level of 0.05 does not prove the absence of an effect but instead suggests that there is insignificant evidence to reject the null hypothesis. Thus, we choose to highlight these findings if they were associated with at least moderate effect sizes to promote future research on these variables, which are necessary to confirm our observations.

Despite the above limitations, results of the current study demonstrate that despite a general hyposensitivity to pain, age- and race-related pain disparities persist among former ASF players as seen in the general population. Moreover, age and race differences in pain sensitivity may be contributing to disparities in clinical pain intensity. It is likely that a host of biological, psychological, and social mechanisms (eg, racial discrimination, exposure to adverse childhood experiences and intergenerational trauma, social determinants of health) could be driving these disparities and should be further examined among former professional athletes to develop interventions to not only address disparities but also protect the health and wellbeing of former athletes, especially those who are older and who belong to racially minoritized groups.

## Disclosures

Dr. Zafonte reported receiving royalties from Springer/Demos Publishing for serving as coeditor of the text Brain Injury Medicine; serving on the scientific advisory board of Myomo Inc., Nano Dx, and onecare.ai Inc.; evaluating patients in the Massachusetts General Hospital Brain and Body–TRUST Program, which is funded by the NFL Players Association; and receiving grants from the NIH. Dr. Taylor reported receiving grants from the NFL Players Association outside the submitted work and grants from the NIH. Dr. Baggish has received funding from the National Institutes of Health/National Heart, Lung, and Blood Institute, the National Football League Players Association (NFLPA), and the American Heart Association and receives compensation for his role as team cardiologist from the US Olympic Committee/US Olympic Training Centers, US Soccer, US Rowing, the New England Patriots, the Boston Bruins, the New England Revolution, and Harvard University. Dr. Weisskopf reported receiving grants from the NFL Players Association and the NIH during the conduct of the study. Drs. Grashow, Whittington and Speizer received grant funding from the NFL Players Association. Mr. Thomas is a member of the Society for Neurosports and serves on the FPHS Player Advisory Board. Dr. Tenforde is Senior editor for PM&R Journal. He gives professional talks such as grand rounds and medical conference plenary lectures and receives honoraria from conference organizers. He has participated in research funded by Arnold P. Gold Foundation (physician and patient care disparities), Football Player Health Study at Harvard (health in American-Style Football players), American Medical Society for Sports Medicine (bone density research), Uniform Health Service and Enovis (Achilles tendinopathy), and MTEC/Department of Defense (bone stress injuries with shockwave). He is a paid consultant for State Farm Insurance and Strava. He receives industry support from Enovis, Sanuwave, and Storz for equipment use for research studies on treatment of tendinopathy, knee osteoarthritis, and bone stress injuries. The remaining authors have no conflict of interest to declare.

## Supplemental digital content

Supplemental digital content associated with this article can be found online at http://links.lww.com/PR9/A395.
