# Supplementary material for: Demographic patterns in quantitative sensory testing and clinical pain among former professional American-style football players
Source: Pain Rep. 2026 Apr 3;11(3):e1428. doi: 10.1097/PR9.0000000000001428 (PMC13052949; doi:10.1097/PR9.0000000000001428)
Supplement: Supplementary file 1 [file painreports-11-e1428-s001.pdf]

Supplemental Methods

We created inverse probability of attrition weights (IPW) for selection into the In-Person Assessment (IPA) subgroup of the Football Players Health Study (FPHS). [4; 13; 14] Briefly, we used a single logistic regression model to predict the probability of selection into the IPA from among all 2,217 FPHS participants eligible for the IPA. Eligibility criteria included age at enrollment survey completion between 28-55 years; reporting 0, 1, or 3 or more of 4 pre-defined afflictions; and self-identified only as Black or White (see Supplemental Material, Table S1). The final IPA study sample included 110 FPHS participants (Table S1). The participation model included a large number of *a priori*–defined probable predictors (Table S2) and used backward selection to inform the variables included in the final models (Table S2). Unstabilized weights were calculated as the inverse of the final probability for each IPA participant being selected. Stabilized weights were calculated by multiplying the unstabilized weights by the marginal probability of enrolling in the IPA (Table S3). We truncated the stabilized weights at the 1st and 99th percentiles such that values lying below or above these thresholds were assigned the 1st and 99th percentile weight respectively.

Supplemental Tables

Table S1. Characteristics of the eligible and final in-person assessment (IPA) cohort.

**Characteristic**	**All IPA Eligible N = 2,217**	**IPA Participants N = 111**
**Age**		
Mean (SD)	42.1 (8.2)	42.9 (7.7)
Min ; Max	28.0 - 55.0	28.0 - 55.0
Median (Q1, Q3)	42.0 (35.0, 50.0)	43.0 (37.0, 49.5)
**Race**		
White	1116 (50.3%)	52 (46.8%)
Black	1101 (49.7%)	59 (53.2%)
**Debut year**		
Mean (SD)	1995 (8.8)	1995 (8.4)
Min ; Max	1969 - 2016	1981 - 2014
Median (Q1, Q3)	1996 (1988, 2003)	1994 (1987.5, 2003)
N-Miss	10	
**Career duration**		
Mean (SD)	6.4 (3.9)	5.6 (3.4)
Min ; Max	1.0 - 25.0	1.0 - 16.0
Median (Q1, Q3)	6.0 (3.0, 9.0)	5.0 (3.0, 7.5)
**Age of first football exposure**		
Mean (SD)	11.0 (3.1)	10.9 (2.8)
Min ; Max	3.0 - 26.0	5.0 - 18.0
Median (Q1, Q3)	11.0 (8.0, 13.0)	11.0 (9.0, 13.0)
N-Miss	19	2
**Current BMI**		
Mean (SD)	31.7 (5.1)	32.5 (5.5)
Min ; Max	19.3 – 57.0	23.5 - 52.5
Median (Q1, Q3)	30.7 (28.1, 34.4)	31.6 (27.9, 35.9)
N-Miss	13	1
**Sleep apnea affliction**		
No	1797 (81.1%)	74 (66.7%)
Yes	420 (18.9%)	37 (33.3%)
**Pain affliction**		
No	1697 (76.5%)	75 (67.6%)
Yes	520 (23.5%)	36 (32.4%)
**Neurocognitive affliction**		
No	1859 (83.9%)	80 (72.1%)

Yes	358 (16.1%)	31 (27.9%)
**Cardiometabolic affliction**		
No	1862 (84.0%)	78 (70.3%)
Yes	355 (16.0%)	33 (29.7%)

Table S2. Variables used in initial and final backward selection logistic regression models to calculate inverse probability of attrition weights (IPW).

**Variable included in initial backwards selection model**	**Included in final model**
ADD/ADHD	
Age at time of enrollment	
Anxiety symptoms	
Cardiometabolic affliction	
Career duration	✓
Concussion signs and symptoms score	
CTE diagnosis from a medical provider	✓
Current anxiety medication	✓
Current BMI	✓
Current depression medication	
Current memory loss medication	✓
Current pain medication	✓
Debut year	✓
Depression symptoms	
Domestic status	✓
Employment status	
Had help completing enrollment questionnaire	
Heart condition (e.g., atrial fibrillation, history of myocardial infarction)	✓
Lineman status	✓
Met US Health & Human Services physical activity standards	✓
Neurocognitive affliction	✓
Number of afflictions	
Number of mid-career surgeries	
Number of post-career surgeries	
Overall health rating	
Overall quality of life rating	✓
Pain intensity	
Race	✓
Received a blast enrollment email invitation	✓

Resided in a northeast or north Atlantic state	✓
Resident of Massachusetts	✓
Responded to recruitment phone call	
Satisfaction with social relationships	
Self-reported dementia diagnosis	
Self-reported diabetes	
Self-reported headaches	
Self-reported high cholesterol	✓
Self-reported hypertension	✓
Self-reported low testosterone	
Self-reported stroke	✓
Sleep apnea affliction	✓
stroke_new_imp1	
Subjective cognitive symptoms	

Table S3. Descriptive statistics for final unstabilized, stabilized and truncated weights

	**Weights N = 111**		
**Statistic**	**Unstabilized**	**Stabilized**	**Truncated**
Mean	8.921	0.853	0.776
Standard deviation	15.455	1.478	0.981
Median	3.195	0.306	0.306
Minimum	1.081	0.103	0.103
1st percentile	1.136	0.109	0.109
5th percentile	1.381	0.132	0.132
10th percentile	1.568	0.15	0.15
90th percentile	21.973	2.101	2.101
95th percentile	30.476	2.914	2.914
99th percentile	47.752	4.565	4.502
Maximum	135.747	12.978	4.565
